# Can Live-Remote Delivery of Supervised Group Exercise Reduce Cancer Health Disparities? Insights from a Community Readiness Assessment and Feasibility Trial of the Exercising Together Program in Underserved Oregon Counties

**DOI:** 10.21203/rs.3.rs-7152633/v1

**Published:** 2025-07-31

**Authors:** Deanne Tibbitts, Christopher Chalmers, Sydnee Stoyles, Jackilen Shannon, Nathan Dieckmann, Karen S. Lyons, Kerri Winters-Stone

**Affiliations:** Oregon Health & Science University; Oregon Health & Science University; Oregon Health & Science University; Oregon Health & Science University; Oregon Health & Science University; Boston College; Oregon Health & Science University

**Keywords:** Rural, Exercise, Dyadic, Health Disparities, Cancer Survivorship

## Abstract

**Background:**

Rural cancer survivors living with poverty and their partners have poorer health outcomes than their urban-dwelling counterparts and encounter barriers to implementing health behaviors that can improve quality of life. We investigated the readiness of geographically underserved (rural with high or persistent poverty) counties in Oregon to engage in health-related research to inform the subsequent conduct of a pilot study to assess the feasibility, acceptability, and preliminary efficacy of a live-remote, supervised dyadic exercise program (Exercising Together^Ó^ (ET)) in couples coping with cancer in those counties.

**Methods:**

We conducted a Community Readiness Assessment in 5 geographically underserved Oregon counties and calculated their readiness scores on a scale from 1 (No awareness) to 9 (Professionalization). The pilot study recruited breast, prostate, and colorectal cancer survivors and their intimate partners living in these 5 counties. Participants were assigned to one of two resistance training programs (ET or unsupervised home-based exercise) for 6 months. Feasibility was assessed by study accrual and retention, completion of surveys and testing visits, intervention adherence, and safety. Participants completed a post-intervention acceptability survey and performance testing and patient-reported outcomes at 0, 6, and 12 months.

**Results:**

Readiness for health-related research varied but was low overall (2.7, Denial/Resistance to 5.2, Preparation). The pilot study enrolled 11 couples (31% accrual), with 73% residing in the highest readiness county. The study had high retention in the ET arm (93%) and high data completeness (surveys, 98%; performance testing visits, 96%), intervention adherence (ET, 93%; unsupervised, home-based exercise, 83%), and safety in both the ET and unsupervised, home-based exercise arms. ET received higher overall experience ratings than unsupervised, home-based exercise, and both survivors and partners in ET experienced large effect-size improvements in physical performance and smaller improvements in some patient-reported outcomes after the exercise program.

**Conclusion:**

Delivering exercise via live-remote classes could address health disparities for rural cancer survivors by increasing access to supervised, evidence-based programs. Since readiness scores were aligned with pilot study enrollment, future studies should prioritize activating low-readiness counties to increase participation in exercise interventions for couples coping with cancer.

**Implications for Cancer Survivors:**

Live-remote exercise classes may increase access to supervised, evidence-based programs for rural cancer survivors.

## INTRODUCTION

Cancer survivors who live in rural America and who live with poverty are more likely to be diagnosed with advanced cancer, to die from cancer, and to have worse quality of life than Americans living in urban areas and/or with higher incomes [1]. While risk factors for rural health disparities include geographic isolation, higher rates of health risk behaviors, limited access to healthcare specialists and subspecialists, and lack of insurance [2], poverty is a particularly potent risk factor [3]. Rural cancer survivors are significantly more likely to report fair/poor health, psychological distress, ≥ 2 non-cancer comorbidities, and health-related unemployment than other survivors [4]. Most cancer survivors are also married when diagnosed, so cancer also threatens the health of the spouse and their relationship [5–7]. Spouses provide most of the supportive care to an ill partner and develop higher rates of hypertension, CVD, obesity, and mortality than spouses who aren’t caregivers [6, 7], and poor health outcomes for caregivers are further compounded by rurality and poverty [8–11]. However, rural cancer survivors and their spouses are the least likely to have access to programs and resources to improve their health and well-being.

Improving health behaviors, such as physical activity, can have a substantial impact on quality of life and survival in cancer survivors, but rural cancer survivors living with poverty are typically inactive [12]. According to the evidence-based exercise guidelines for cancer survivors, many cancer-related health outcomes, including fatigue, anxiety, depression, physical functioning, and health-related quality of life, can be improved with modest amounts of moderate intensity exercise just two to three times per week [13]. Spouse/partners can also benefit from regular exercise to counter the adverse impact of caregiving on physical and mental health [14, 15]. Benefits from exercise are usually greater when programs are supervised, likely due to support from trainers and peers that leads to better retention, adherence and motivation [16]. Unfortunately, survivors living in rural areas and/or with poverty face many barriers to exercise, especially facility-based, supervised exercise programs [17, 18]. Although a few trials have tested the benefits of exercise in rural cancer survivors, most programs rely on web-based or mail-based independent exercise, often lacking the additional support provided by coaches and peers [19] and resulting in lower likelihood of meeting exercise goals [20]. While there are no physical activity interventions for spouses of cancer survivors living in rural areas, it is also likely that they will be prone to inactivity. Rural cancer survivors report improvements in cancer-related health outcomes from supervised group exercise programs [17, 21]; however, to increase participation, exercise interventions need to address the barriers that rural cancer survivors and their spouses experience, such as cost, transportation, and awareness of exercise programs [17, 18].

We have developed, tested, and adapted a partnered exercise program that could address barriers to exercise experienced by couples coping with cancer who reside in rural areas with high or persistent poverty (referred to as geographically underserved areas [22]). Exercising Together^©^ is a resistance training program that aims to improve the physical and mental health of the cancer survivor, their spouse, and the health of their relationship by having them exercise as a team [23]. Our large, randomized trial of Exercising Together^©^ [23] was interrupted by the COVID-19 pandemic and required us to adapt exercise delivery to a live-remote format using video conferencing software [24]. Exercising Together^©^ is delivered in a supervised, group format, even online, providing additional social support for couples. These adaptations to Exercising Together^©^ could make the program more accessible to cancer survivors and their spouses living in geographically underserved areas by removing access barriers yet retaining elements of supervised exercise that optimize exercise benefits. However, successful implementation of this partnered program requires that we assess both the feasibility of the program in our target communities and each community’s readiness to engage in health-related research, as readiness is an important contextual factor that has been shown to affect the success of health-related interventions [25–29].

The purpose of this study was to understand the readiness of geographically underserved counties in Oregon to engage in health-related research and to conduct a pilot feasibility study of live-remote delivery of Exercising Together^©^ to couples coping with cancer in those counties. We also assessed the acceptability of the program and examined changes in cancer-related health outcomes during and after the exercise program.

## METHODS

### Design and Setting

**Figure 1:**
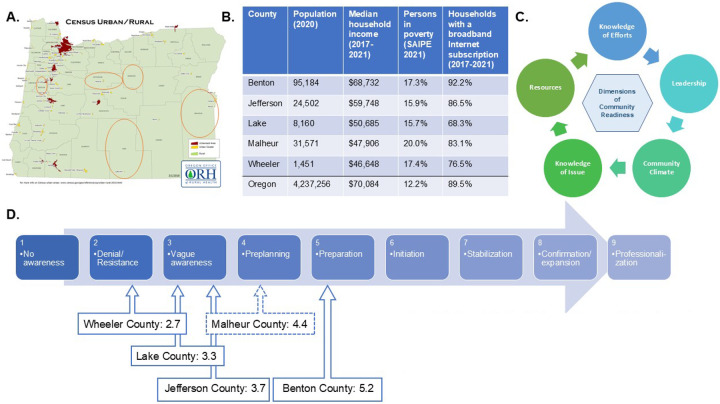
Readiness for health-related research in geographically underserved counties in Oregon. **(A)** Map of Oregon showing the location of the five geographically underserved counties (orange circles). **(B)** Population, income level, and internet access in Oregon’s five geographically underserved counties (statistics from the US Census bureau) and Oregon overall. **(C)** Community Readiness Assessment model showing the five dimensions of community readiness assessed through interviews and focus groups with key respondents. **(D)** Overall readiness score for health-related research by county. Readiness is scored from 1–9, with higher scores indicating greater readiness. Note: Overall readiness score for Malheur County is based on n=1 stakeholder interview. Map in (A) is adapted from the Oregon Office of Rural Health. SAIPE, Small Area Income and Poverty Insurance Estimates.

We conducted a Community Readiness Assessment (CRA) followed by a feasibility study. The purpose of the CRA was to determine the level of readiness to engage in health-related research within geographically underserved counties [22] (defined as counties with high and/or persistent poverty in Frontier and Remote (FAR) area zip codes). Oregon contains five counties that meet criteria for being geographically underserved (Fig. 1). Two counties are located in central Oregon (Jefferson, Wheeler), two in southeastern Oregon (Lake, Malheur), and one in western Oregon (Benton) (Fig. 1A). Central and southeastern counties have populations ranging from 1,451–31,571, median household incomes between 67% and 85% of the state’s median income, and percent in poverty ranging from 15.7% − 20.0% (Fig. 1B). While Benton County meets the criteria for being geographically underserved, it contains a state university and has a larger population (95,184) and median income ($68,732, comparable to the state average), which distinguish it from other geographically underserved counties in Oregon.

Following the CRA, we conducted an ancillary feasibility study to the Exercising Together^©^ trial (NCT03630354) [23], with the intention to recruit 30 couples and randomly assign them to the three study arms: Exercising Together^©^, a partnered exercise program (Arm 1); Separate Survivor and Spouse/Partner classes, where survivors and spouses/partners perform exercise routines with other survivors or spouse/partners, respectively (Arm 2); or an unsupervised, home-based exercise program where couples are taught a similar training program as Arm 2 that is done independently at home (Arm 3). Due to low initial enrollment and limited time and resources, the study design was changed to a quasi-experimental design of Arms 1 and 3; couples were recruited to participate in Arm 1, but if they were unwilling or unable to participate in a structured group exercise program, they were given the option to participate in Arm 3. This design allowed us to first assess feasibility of Exercising Together^©^, the experimental arm, but also to determine if unsupervised exercise was a feasible alternative approach. All participants engaged in the study arms for 6 months and were followed up 6 months later.

The CRA and ancillary feasibility study were conducted remotely by staff at Oregon Health & Science University in Portland, Oregon. The OHSU Institutional Review Board (IRB) approved the feasibility study (IRB#18000), and all participants gave written informed consent prior to baseline data collection.

### Community Readiness Assessment

The CRA [30] includes identifying key respondents (stakeholders) that represent different sectors of the community and interviewing key respondents with 35 questions structured around five dimensions of readiness: *knowledge of issue, knowledge of efforts, community climate, leadership, and resources* (Fig. 1C). Our CRA assessed readiness to participate in health-related research among stakeholders in five geographically underserved counties in Oregon. The CRA team conducted phone or video interviews to inquire about communities’ attitudes, knowledge, and beliefs about participating in health-related research. Transcripts were grouped by county and analyzed to create a score for each dimension of readiness and an overall score, ranging from 1 (No awareness) to 9 (Professionalization). The overall score then guides recommendations for next steps for the research team to work within each community.

### Feasibility Study

#### Sample and Recruitment

The sample for the feasibility study was breast, prostate, or colorectal cancer survivors and their co-residing intimate partners. Eligibility was mostly similar to the parent trial, which has been described elsewhere [23], plus currently residing in one of five geographically underserved counties in Oregon (Wheeler, Malheur, Jefferson, Lake, and Benton) and widened criterion for time since diagnosis from 3 to 10 years to improve the recruitment pool and yet still enroll couples who may be coping with persistent impacts of cancer and treatment. Participants without internet access or videoconference-capable devices would be provided with a laptop or cellular-enabled tablet if needed. We aimed to enroll 10 couples per study arm.

Recruitment procedures included engaging with clinics in our target counties, local contacts from the CRA, the Oregon Office of Rural Health, and the Oregon Rural Practice-based Research Network to share information about the study with their community. We also directly mailed study invitation letters to potentially eligible cancer survivors who resided in a target county and who were identified using the Oregon State Cancer Registry (n = 849) or the OHSU Cancer Registry (n = 66). Some OHSU registry patients also received a MyChart message. We also ran social media ads targeted at adults living in our counties of interest. Interested couples were able to share their contact information with the community partners, email the study team, mail back the study interest forms, or directly communicate their interest by MyChart.

#### Procedures

Interested couples were screened by research staff over the phone, including evaluation of their video conferencing capabilities (internet, device, experience). Participants were offered internet or device assistance as needed. For eligible couples, the study consent form was emailed and signed electronically. Couples who declined to participate in the live-remote study arm (due to scheduling conflicts, etc.) were offered the option of participating in the unsupervised, home-based study arm. All couples completed baseline testing via videoconference before beginning their exercise program. Outcome measures were collected at 0 (baseline), 6 (post-intervention), and 12 months (6 months after completion of the intervention). Since this was a feasibility study with limited resources, assessors were not blinded to group assignment.

#### Study Intervention

Details of the exercise interventions are published [23]. All participants were expected to engage in two 1-hour Exercising Together^©^ sessions per week for 6 months. For Exercising Together^©^ (Arm 1), exercise classes were delivered in a live-remote format via videoconference following our protocol for remote delivery [24]. For the unsupervised, home-based program (Arm 3), participants met virtually with exercise instructors twice over a 1–2 week period to learn the study exercise program and received a pre-recorded exercise video and written training plan to complete on their own. Exercise equipment (steps and risers, weighted vests, and adjustable dumbbells) was shipped to all participants.

#### Six-Month Follow-Up Period

To evaluate whether benefits gained from exercise persisted outside of the planned programs, couples were followed for an additional 6 months after formal training stopped. We provided a partnered exercise video to stream at home for couples in the Exercising Together^**©**^ arm, while the unsupervised, home-based arm could continue to use their video as desired.

#### Study Measures

##### Accrual, Retention, Data Completeness, Adherence, and Safety

Feasibility was assessed by study accrual and retention, completion of surveys and testing visits, intervention adherence, and safety. Accrual was defined as the number of participants who enrolled and received an intervention out of the number of people approached for the study. Retention was defined as the number of participants who remained enrolled in the study for 12 months out of all participants who enrolled and received the intervention. Survey and testing visit data completeness was assessed by the proportion of surveys and testing visits completed out of the prescribed time points. Intervention adherence was assessed by the median number of exercise sessions completed. Intervention safety was assessed by monthly adverse event reporting using an emailed survey and was categorized as mild, moderate or serious and related, possibly related or not related to the study by a member of the study team.

##### Acceptability

Acceptability was assessed with a survey sent at the end of the 6-month intervention period.

Participants were asked to rate their overall experience, the accessibility, effectiveness, and enjoyment of their assigned exercise program. Overall experience was rated using a 10-point scale, where 1 = Poor, 5 = Acceptable, and 10 = Exceptional; accessibility, effectiveness, and enjoyment were rated using a 4-point Likert scale (1 = Strongly disagree, 4 = Strongly agree). Participants in the Exercising Together^©^ arm were also asked if they preferred a live-remote versus in-person class setting, and they were asked to rate their experience with the live-remote format using a 4-point Likert scale (1 = Strongly disagree, 4 = Strongly agree).

##### Objectively Measured Outcomes

Physical performance was assessed during a videoconference using protocols adapted for remote assessment [23, 31]. Upper body strength was assessed by a push-up test by recording the number of standard or modified (knee) push-ups completed in one continuous bout until fatigue (i.e., loss of form and/or stopping) [32]. Core strength was assessed with a timed plank test, which recorded how long participants could hold a standard or modified (knee) plank position until fatigue. The short Physical Performance Battery (sPPB) consists of an aggregated score based on performance in standing balance, 4-meter usual walk, and 5-time sit to stand tests [33]. Sit to stand times were also used as an independent measure of lower extremity strength.

##### Patient-Reported Outcomes

Patient-reported physical and mental health were measured by the SF-36 Medical Outcomes Survey [34] physical and mental composite scores. Scores range from 0–100, with higher scores indicating better health. We evaluated anxiety using the PROMIS Anxiety Short Form 8a [35]; raw scores are converted to a standardized T-score (mean = 50, SD = 10), and higher scores indicate more anxiety. We evaluated depressive symptoms using the Center for Epidemiologic Studies Depression Scale (CES-D [36]); scores range from 0–60, with higher scores indicating greater depressive symptoms.

### Statistical Analysis

Feasibility and acceptability outcomes were described with standard descriptive statistics (mean +/− standard deviation and percentages). We also examined within-group changes in objective and patient-reported outcomes in the Exercising Together^©^ arm by calculating the mean difference in outcomes over the intervention (0–6 months) and follow up (6–12 months) periods. We calculated effect sizes (Cohen’s d) for within-group changes in study outcomes. Effect size interpretation was based on Sawilowsky’s 2009 recommendations [37]. We did not examine within-group changes in the unsupervised, home-based arm due to an insufficient sample size with pre-post outcomes (n = 2). Descriptive statistics, within-group changes, and effect size calculations were conducted using R version 4.3.2 [38].

## RESULTS

### Community Readiness Assessment

The CRA was implemented by interviewing 14 stakeholders across five counties (Fig. 1A, B): Benton (n = 4), Jefferson (n = 3), Lake (n = 3), Malheur (n = 1), and Wheeler (n = 3) and included both stakeholder focus groups and individual stakeholder interviews. The five counties varied in their level of readiness to engage in health-related research, with Benton County having the highest level of readiness (score 5.2 = “Preparation”). Malheur County had the next highest level of readiness (4.4; “Pre-planning”), but was based on limited stakeholder involvement compared to other counties. Two of the counties scored as having “Vague Awareness” of health-related research (Jefferson, 3.7 and Lake, 3.3), and one as having “Denial/Resistance” (Wheeler, 2.7) (Fig. 1D).

### Feasibility Study

#### Sample and Recruitment

**Figure 2:**
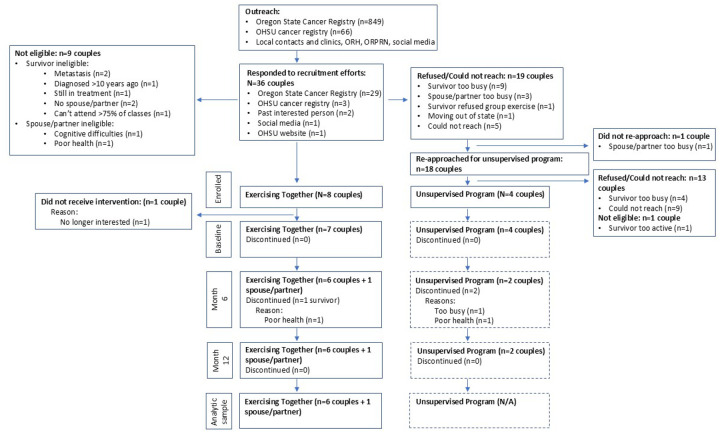
CONSORT diagram of participant flow through the feasibility trial.

Thirty-six couples from four of the five target counties responded to recruitment efforts (Fig. 2), and 12 couples enrolled into one of the two study arms, though one couple withdrew before completing all baseline measures, yielding a final sample of n = 11 and a 31% accrual rate among interested couples. Eight couples initially enrolled in the Exercising Together^©^ arm, and of the 18 couples we reapproached, four enrolled in the unsupervised, home-based program. No couples needed assistance with internet access or needed an internet-enabled device.

Enrolled survivors and partners were an average of 66 years old, partnered for an average of 33 years, mostly college educated (64% of survivors, 82% of partners), and on average had a household income of at least $50,000/year (71%) (Table 1). Most couples resided in Benton County (n = 8; 73%), with remaining couples residing in Jefferson (n = 1), Lake (n = 1), and Wheeler (n = 1) counties; no couples enrolled from Malheur County. Enrolled survivors were diagnosed with breast cancer (91%) or colorectal cancer (9%) and were an average of 4.4 years post-diagnosis.

#### Retention, Data Completeness, Adherence, and Safety

Study retention was 77%, with higher retention in the Exercising Together^©^ arm (93%) than the unsupervised, home-based arm (50%). During the 6-month intervention period, one survivor dropped out of the Exercising Together^©^ arm due to poor health, though their spouse continued to participate in the intervention, and two couples dropped out of the unsupervised, home-based arm due to poor health (n = 1) or being too busy (n = 1) (Fig. 2). Participants completed 98% of all surveys (100%, Exercising Together^©^ arm; 94%, unsupervised, home-based arm) and 96% of all testing visits (100%, Exercising Together^©^ arm; 88%, unsupervised, home-based arm). Exercise adherence was higher in the Exercising Together^©^ arm (median attendance: 93%) than the unsupervised, home-based arm (median completion of prescribed sessions: 83%). No moderate or serious adverse events related to the study were reported during the 12-month study period.

#### Acceptability

Participants who completed the study programs reported a positive overall experience, with couples in the Exercising Together^©^ arm rating their experience “Exceptional” (median of 10 out of 10 for both survivors and partners), which was higher than the unsupervised, home-based program rating of above “Acceptable” (median of 6.5 and 7.0 out of 10 for survivors and partners, respectively) (Supplementary table 1). Most survivors and partners in the Exercising Together^©^ arm reported a preference for a live-remote exercise class over an in-person class (83% of survivors and 57% of partners). Additional acceptability survey data are available in Supplementary Table 1.

#### Objectively Measured Outcomes

Survivors and partners in the Exercising Together^©^ arm improved on several physical performance measures with large to very large effect sizes, particularly for survivors (Supplementary Table 2). Survivors increased their upper body strength (d = 7.2), core strength (d = 1.8), physical functioning (d = 1.1), and lower body strength (d = 1.8) over the 6-month intervention. During follow-up, survivors maintained improvements in core strength relative to the post-intervention timepoint; other physical performance measures deteriorated during follow-up with very small to medium effect sizes but remained improved relative to baseline values.

Partners also increased their upper body strength (d = 0.9), core strength (d = 1.5), physical functioning (d = 0.5) and lower body strength (d = 0.9) over the 6-month intervention. During follow-up, partners maintained improvements in physical functioning relative to the post-intervention timepoint and exhibited slight deterioration in other performance measures, but these remained improved relative to their baseline values.

#### Patient-Reported Outcomes

Survivors in the Exercising Together^©^ arm reported improvements in physical and mental health composite scores with small effect sizes (d = 0.2; d = 0.2) over the intervention period and further improvement in physical health composite score with a small effect size (d = 0.4) during follow-up. Survivors reported a decrease in anxiety over the intervention with a very large effect size (d = 1.9), which then partially reversed during the 6-month follow-up period with a large effect size (d = 1.0), though anxiety remained lower than baseline (Supplementary Table 2). Survivors’ depressive symptoms were stable across all timepoints. Partners in the Exercising Together^©^ arm reported improvements in physical health composite score with a small effect size (d = 0.2) over the intervention and a decline in physical health composite score with a very small effect size (d = 0.1) over the follow-up period, remaining improved relative to baseline. Partners reported no change or very small effect sizes for changes in mental health composite score, anxiety, and depressive symptoms over the intervention and follow-up periods.

## DISCUSSION

With the widespread shift to live-remote exercise delivery and performance assessment in response to the COVID-19 pandemic, we had a unique opportunity to investigate the readiness for health-related research and feasibility of a supervised group exercise program aimed to help couples coping with cancer living in geographically underserved counties in Oregon. We found that readiness for health-related research varied but was low overall. Accordingly, we were challenged by low accrual into our pilot study, but there was high retention, data completeness, adherence, safety, and acceptability to live-remote, supervised group exercise. Unsupervised, home-based exercise also had strong feasibility but had consistently lower satisfaction ratings. Both survivors and partners in Exercising Together^©^ experienced large effect-size improvements in physical performance and smaller improvements in some patient-reported outcomes in response to the exercise program. Gains from exercise mostly declined over a six-month follow-up period after structured classes ended but still remained above baseline levels.

While readiness for health-related research was low overall in geographically underserved counties, one (Benton County) scored in the middle of the readiness scale (“Preparation”), suggesting that not all counties would need the same approach to improving readiness for health research. For counties scoring in the “Denial/Resistance” and “Vague Awareness” categories, the CRA-recommended next steps included visiting local leaders and community groups, collecting and disseminating stories from local people who have experience related to the health topic of study, and hosting events to increase awareness of what health-related research is and how it connects to local needs. For Benton County (“Preparation”), the CRA recommendations assume that residents are already aware of how health-related research connects to local needs and instead recommend conducting public forums to develop strategies to advance health-related research and advertising with community leaders or support groups. Interestingly, a key factor in Benton County’s designation as geographically underserved is the presence of a university, which contributes to a disproportionately high number of residents aged 18–24 [39], most of whom live below the federal poverty level. This unique demographic feature may contribute to the difference in readiness between Benton County and the other geographically underserved counties in Oregon. Since the majority of the feasibility study sample were residents of Benton County, our future efforts to improve readiness for research should preferentially target counties with lower readiness, making the CRA a valuable tool to guide resource allocation for future community engagement.

Among enrolled couples, feasibility of the Exercising Together^©^ pilot study was very strong, with high retention, data completeness, adherence, and safety, coupled with high acceptability and improvements in physical performance and some patient-reported outcomes among couples participating in the Exercising Together^©^ program. We observed that the level of readiness in each county corresponded to the amount of enrollment from each county into the feasibility study, with the highest number of couples enrolling from the county with the highest readiness and one or no couples enrolling from each of the counties with lower readiness. Community activation around an issue has been previously shown to affect intervention success, but there has been limited use of tools like a CRA to help plan research aimed to study how to widen the reach of exercise programs to communities with high need but poor access to optimal programs. In prior studies, health-related interventions had greater participation and were more successful at improving outcomes when a community exhibited higher levels of readiness [25, 26, 29]. Thus, in our study, activating low-readiness counties by implementing recommendations from the CRA may have improved enrollment and should be a focus of future efforts to increase participation in exercise interventions for couples coping with cancer.

Rural cancer survivors who participated in Exercising Together^©^ demonstrated improvements in physical functioning and strength that were consistent with improvements observed in a larger trial [40] of this intervention. Objective measures of upper and lower body strength—such as push-ups, planks, and chair stands—as well as a patient-reported measure of anxiety improved with large to very large effect sizes, suggesting that rural survivors can achieve meaningful benefits to their physical and mental health when provided with a structured, accessible intervention. In addition, Exercising Together^©^, delivered as a live-remote intervention, received a higher overall experience rating versus the unsupervised, home-based program, though both interventions received positive ratings overall. Delivering exercise via live-remote classes may offer a promising strategy to reduce health disparities between rural and urban cancer survivors by increasing access to supervised, evidence-based programs.

This study had several strengths, but also limitations. This is one of the first studies to assess the readiness of rural Oregon counties to participate in health-related research and to implement a live-remote exercise intervention for cancer survivors and their partners in those communities. This pilot project demonstrates that live-remote, exercise-based interventions are not only acceptable but also show similar efficacy to larger clinical trials conducted in urban or higher-resourced settings. This is of significant value to rural communities, which often lack the infrastructure and personnel to support in-person delivery of such programs. Moreover, it highlights the potential scalability of remote interventions to overcome long-standing barriers to research participation, expanding access for both rural populations and the researchers working to support them. While the CRA process generated recommendations for increasing community readiness for health-related research, due to time and resources constraints we were unable to implement these recommendations prior to initiating recruitment for the feasibility study. We were also not able to robustly evaluate the readiness of Malheur County due to the low number of stakeholders who engaged with the CRA. Most participants in the feasibility study reported an annual income above $50,000, which limits our understanding of program feasibility for survivors and partners experiencing higher poverty levels. We were not able to evaluate the benefits of the unsupervised, home-based exercise program due to low sample size, and thus we lacked a control group to compare changes observed from the Exercising Together^©^ program. Finally, we did not assess how much participants used their exercise video during the 6-month follow-up period and thus do not know whether continuation of the program contributed to maintenance of or changes in outcomes across that period.

Our results underscore the importance of community readiness for the success of interventions, such as supervised group exercise programs, to address health disparities in geographically underserved cancer survivors and caregivers. With the development of live-remote exercise program delivery [24], cancer survivors and caregivers can be reached with supervised group exercise programs that can address known barriers to exercise in rural communities, including cost and transportation [17, 18]. However, for survivors and caregivers living in rural areas to benefit from these programs, it is crucial to lay the groundwork that will increase their participation. Conducting a Community Readiness Assessment is thus a key step in understanding a community’s capacity to engage in health-related research, identifying the barriers to successful intervention, and building receptivity to interventions that can improve cancer survivorship outcomes. Future exercise studies should therefore build in time to implement readiness-increasing recommendations well in advance of recruitment in order to maximally reach survivors and caregivers living in geographically underserved areas. With the increasing decentralization of clinical trials, efforts to increase readiness for health-related research in these communities could also globally increase their participation in interventional studies to address cancer-related health disparities.

## Supplementary Material

Supplementary Files

This is a list of supplementary files associated with this preprint. Click to download.

- SupplementaryInformationGEOtables.docx

## Figures and Tables

**Table 1:** Demographic characteristics of the sample.

	Whole Sample		Exercising Together		Unsupervised Home-Based Program	
	Survivor	Partner	Survivor	Partner	Survivor	Partner
	(N = 11)	(N = 11)	(n = 7)	(n = 7)	(n = 4)	(n = 4)
Characteristic	M (SD) or %	M (SD) or %	M (SD) or %	M (SD) or %	M (SD) or %	M (SD) or %
Age	66.1 (7.9)	66.4 (5.7)	66.1 (10.18)	67.4 (5.4)	66.2 (0.4)	64.7 (6.8)
Gender						
Male	9%	91%	14%	86%	0%	100%
Female	91%	9%	86%	14%	100%	0%
Race (White/Caucasian)	100%	100%	100%	100%	100%	100%
Ethnicity (Non-Hispanic)	100%	100%	100%	100%	100%	100%
Education (Bachelors or higher)	64%	82%	71%	71%	50%	100%
Employment outside home	27%	27%	29%	14%	25%	50%
Income						
$25,000–49,999	18%	18%	29%	29%	0%	0%
$50,000–99,999	27%	36%	14%	29%	50%	50%
$100,000–149,999	45%	27%	43%	29%	50%	25%
$150,000 or more	0%	9%	0%	14%	0%	0%
Declined	9%	9%	14%	0%	0%	25%
County						
Benton	73%	73%	71%	71%	75%	75%
Jefferson	9%	9%	0%	0%	25%	25%
Lake	9%	9%	14%	14%	0%	0%
Malheur	0%	0%	0%	0%	0%	0%
Wheeler	9%	9%	14%	14%	0%	0%
Relationship length (years)	33.4 (16.0)	33.1 (15.8)	30.5 (17.8)	29.8 (17.2)	38.5 (12.7)	39.0 (13.0)
Charlson Comorbidity Index^a^	1.8 (0.9)	1.2 (1.6)	1.6 (1.0)	1.4 (1.8)	2.3 (0.5)	0.7 (0.6)
BMI	27.4 (5.5)	26.8 (3.3)	29.2 (6.1)	26.6 (2.6)	24.4 (2.3)	27.3 (4.7)
Cancer type						
Breast	91%		86%		100%	
Colorectal	9%		14%		0%	
Time since diagnosis (months)^b^	53.3 (8.7)		49.2 (7.3)		61.7 (3.5)	
Stage						
0	27%		14%		50%	
I	36%		57%		0%	
II	0%		0%		0%	
III	18%		14%		25%	
No stage	9%		14%		0%	
Don’t know	9%		0%		25%	
Spread	9%		14%		0%	
Treatments						
Surgery	100%		100%		100%	
Radiation	64%		57%		75%	
Chemotherapy	27%		29%		25%	
Hormone therapy	55%		57%		50%	

a missing 1 partner

b n = 9

## Data Availability

The data underlying this article cannot be shared publicly due to the privacy of individuals that participated in the study. Deidentified data are available from the corresponding author upon request through a data use agreement for specific, approved analyses.

## References

[1] Singh GK, Socioeconomic, Rural-Urban, and Racial Inequalities in US Cancer Mortality: Part I-All Cancers and Lung Cancer and Part II-Colorectal, Prostate, Breast, and Cervical Cancers. J cancer Epidemiol. 2011;2011:107497. 10.1155/2011/107497. 22496688 PMC3307012

[2] Sloane D. Cancer epidemiology in the United States: racial, social, and economic factors. Methods Mol Biol. 2009;471:65–83. 10.1007/978-1-59745-416-2_4. 19109775

[3] Long AS, Hanlon AL, Pellegrin KL. Socioeconomic variables explain rural disparities in US mortality rates: Implications for rural health research and policy. SSM Popul Health. 2018;6:72–4. 10.1016/j.ssmph.2018.08.009. 30225336 PMC6138992

[4] Weaver KE, Rural-urban disparities in health status among US cancer survivors. Cancer. 2013;119(5):1050–7. 10.1002/cncr.27840. 23096263 PMC3679645

[5] Baade PD, Fritschi L, Eakin EG. Non-cancer mortality among people diagnosed with cancer (Australia). Cancer Causes Control. 2006;17(3):287–97. 10.1007/s10552-005-0530-0. 16489536

[6] Vitaliano PP, Zhang J, Scanlan JM. Is caregiving hazardous to one’s physical health? A meta-analysis. Psychol Bull. 2003;129(6):946–72. 10.1037/0033-2909.129.6.946. 14599289

[7] Fredman L, Leisure-time exercise and overall physical activity in older women caregivers and non-caregivers from the Caregiver-SOF Study. Prev Med. 2006;43(3):226–9. 10.1016/j.ypmed.2006.04.009. 16737731

[8] Cohen SA, Nash CC, Greaney ML. Place-based, intersectional variation in caregiving patterns and health outcomes among informal caregivers in the United States. Front Public Health. 2024;12:1423457. 10.3389/fpubh.2024.1423457. 39224561 PMC11366647

[9] Sanford JT, Townsend-Rocchiccioli J. The perceived health of rural caregivers. Geriatr Nurs. 2004;25(3):145–8. 10.1016/j.gerinurse.2004.04.007. 15197373

[10] Pinquart M, Sorensen S. Correlates of physical health of informal caregivers: a meta-analysis. J Gerontol B Psychol Sci Soc Sci. 2007;62(2):P126–37. 10.1093/geronb/62.2.p126. 17379673

[11] Li Q, Loke AY. A spectrum of hidden morbidities among spousal caregivers for patients with cancer, and differences between the genders: a review of the literature. Eur J Oncol Nurs. 2013;17(5):578–87. 10.1016/j.ejon.2013.01.007. 23465710

[12] Mama SK, Rural-urban differences in meeting physical activity recommendations and health status in cancer survivors in central Pennsylvania. Support Care Cancer. 2020. 10.1007/s00520-020-05342-y. PMC7415488 32036469

[13] Campbell KL, Exercise Guidelines for Cancer Survivors: Consensus Statement from International Multidisciplinary Roundtable. Med Sci Sports Exerc. 2019;51(11):2375–90. 10.1249/MSS.0000000000002116. 31626055 PMC8576825

[14] Cardoso C, Lumini MJ, Martins T. Effects of physical exercise in reducing caregivers burden: a systematic review. Front Public Health. 2025;13:1474913. 10.3389/fpubh.2025.1474913. 39975791 PMC11836036

[15] Lambert SD, A Descriptive Systematic Review of Physical Activity Interventions for Caregivers: Effects on Caregivers’ and Care Recipients’ Psychosocial Outcomes, Physical Activity Levels, and Physical Health. Ann Behav Med. 2016;50(6):907–19. 10.1007/s12160-016-9819-3. 27439530

[16] Westphal T, Supervised versus autonomous exercise training in breast cancer patients: A multicenter randomized clinical trial. Cancer Med. 2018;7(12):5962–72. 10.1002/cam4.1851. 30415507 PMC6308077

[17] Hirko KA, Implementation of Physical Activity Programs for Rural Cancer Survivors: Challenges and Opportunities. Int J Environ Res Public Health. 2021;18(24):12909. 10.3390/ijerph182412909. 34948517 PMC8702182

[18] Smith-Turchyn J, Characterizing the Exercise Behaviour, Preferences, Barriers, and Facilitators of Cancer Survivors in a Rural Canadian Community: A Cross-Sectional Survey. Curr Oncol. 2021;28(4):3172–87. 10.3390/curroncol28040276. 34436042 PMC8395505

[19] Frensham LJ, Parfitt G, Dollman J. Effect of a 12-Week Online Walking Intervention on Health and Quality of Life in Cancer Survivors: A Quasi-Randomized Controlled Trial. Int J Environ Res Public Health. 2018;15(10). 10.3390/ijerph15102081. PMC6210292 30248943

[20] Gray MS, Rural-urban differences in health behaviors and outcomes among older, overweight, long-term cancer survivors in the RENEW randomized control trial. Cancer Causes Control. 2019;30(4):301–9. 10.1007/s10552-019-01141-x. 30783858 PMC6459722

[21] Gell NM, Remotely delivered exercise to older rural cancer survivors: a randomized controlled pilot trial. J Cancer Surviv. 2024;18(2):596–605. 10.1007/s11764-022-01292-y. 36374436 PMC9662104

[22] Geographically Underserved Areas. 10/24/2024; Available from: https://cancercontrol.cancer.gov/hdhe/research-emphasis/underserved-areas

[23] Winters-Stone KM, Study protocol for the Exercising Together© trial: a randomized, controlled trial of partnered exercise for couples coping with cancer. Trials. 2021;22(1):1–16. 10.1186/s13063-021-05548-3. 34461975 PMC8404361

[24] Winters-Stone KM, Delivering exercise medicine to cancer survivors: has COVID-19 shifted the landscape for how and who can be reached with supervised group exercise? Support Care Cancer. 2022;30(3):1903–6. 10.1007/s00520-021-06669-w. 34741653 PMC8571667

[25] Ehlers DK, Huberty JL, Beseler CL. Is school community readiness related to physical activity before and after the Ready for Recess intervention? Health Educ Res. 2013;28(2):192–204. 10.1093/her/cys102. 23107932

[26] Lawsin CR, Community readiness to promote Latinas’ participation in breast cancer prevention clinical trials. Health Soc Care Community. 2007;15(4):369–78. 10.1111/j.1365-2524.2007.00695.x. 17578398

[27] Aboud F, An assessment of community readiness for HIV/AIDS preventive interventions in rural Bangladesh. Soc Sci Med. 2010;70(3):360–7. 10.1016/j.socscimed.2009.10.011. 19892453

[28] Millar L, Increasing community capacity and decreasing prevalence of overweight and obesity in a community based intervention among Australian adolescents. Prev Med. 2013;56(6):379–84. 10.1016/j.ypmed.2013.02.020. 23485797

[29] Stallones L, Community readiness and prevention of traumatic brain injury. Brain Inj. 2008;22:7–8. 10.1080/02699050802132487. 18568708

[30] Oetting ER. Community Readiness for Community Change. 2 ed. 2014, Fort Collins, CO. 71. https://tec.colostate.edu/wp-content/uploads/2018/04/CR_Handbook_8-3-15.pdf

[31] Guidarelli C, Remote administration of physical performance tests among persons with and without a cancer history: Establishing reliability and agreement with in-person assessment. J Geriatr Oncol. 2022;13(5):691–7. 10.1016/j.jgo.2022.02.002. 35177378 PMC9232927

[32] Chalmers C, Guidarelli C, Stoyles S. Validity, Sensitivity, And Reliability Of Remotely Administered Push-up And Plank Tests. in International Journal of Exercise Science: Conference Proceedings. 2023. https://digitalcommons.wku.edu/ijesab/vol8/iss11/6/

[33] Guralnik JM, A short physical performance battery assessing lower extremity function: association with self-reported disability and prediction of mortality and nursing home admission. J Gerontol. 1994;49(2):M85–94. 10.1093/geronj/49.2.m85. 8126356

[34] Jr W. The MOS 36-item short-form health survey (SF-36). Med Care, 1992. 30(6): p. 473–83, https://pubmed.ncbi.nlm.nih.gov/1593914/1593914

[35] Pilkonis PA, Item banks for measuring emotional distress from the Patient-Reported Outcomes Measurement Information System (PROMIS(R)): depression, anxiety, and anger. Assessment. 2011;18(3):263–83. 10.1177/1073191111411667. 21697139 PMC3153635

[36] Radloff LS. The CES-D scale: A self-report depression scale for research in the general population. Appl Psychol Meas. 1977;1(3):385–401. 10.1177/014662167700100306.

[37] Sawilowsky SS. New effect size rules of thumb. J Mod Appl Stat methods. 2009;8(2):26. 10.56801/10.56801/v8.i.452.

[38] R-Core-Team R. A Language and Environment for Statistical Computing. R Foundation for Statistical Computing. Vienna, Austria; 2023. https://www.r-project.org/.

[39] Banwarth P, Assessment BCCH, Department BCH. Editor. 2021, Benton County Health Department: Corvallis Oregon. pp. 54–55. https://health.bentoncountyor.gov/wp-content/uploads/2023/11/benton_cha_2017_final_11_7_2017.pdf

[40] Winters-Stone KM, Benefits of partnered strength training for prostate cancer survivors and spouses: results from a randomized controlled trial of the Exercising Together project. J Cancer Surviv. 2016;10(4):633–44. 10.1007/s11764-015-0509-0. 26715587

